# Grading diabetic retinopathy: an introduction

**Published:** 2023-07-07

**Authors:** Covadonga Bascaran

**Affiliations:** 1 Clinical Research Fellow, London School of Hygiene and Tropical Medicine, UK.

**The retina can be visualised to check for signs of diabetic retinopathy that require treatment.**

**Figure:**
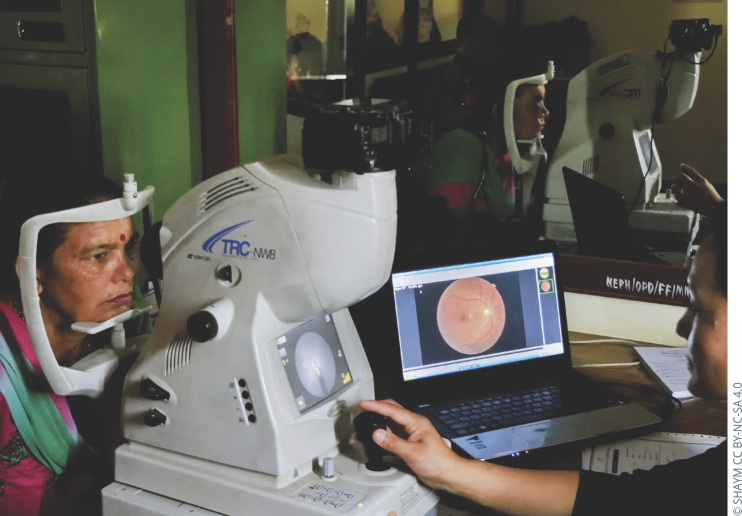
Diabetic retinopathy screening. **NEPAL**

**Figure 1:**
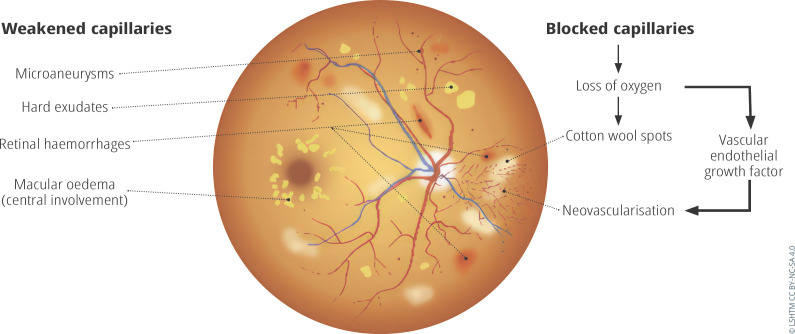
Signs of diabetic retinopathy.

In diabetes, elevated blood sugar levels damage the capillaries in the retina, resulting in a range of different, visible changes (Figure 1), collectively known as diabetic retinopathy (DR).

- **Weakened capillary walls** can result in microaneurysms. Further weakening causes capillaries to leak, causing retinal haemorrhages and the formation of hard exudates.
- **Blocked capillaries** starve the surrounding tissues of oxygen, which results in cotton wool spots and and stimulates the production of vascular endothelial growth factor (VEGF), responsible for the growth of new vessels (neovascularisation).

People living with diabetes rarely notice changes in their vision during the early stages of DR. In order to detect early signs (such as cotton wool spots and microaneuysms), yearly retinal examinations are essential.

The most common method of retinal screening is retinal photography. Trained graders examine the retinal images, looking for signs of DR. These signs are used to classify the level of disease severity and determine whether there is need to refer the patient for treatment. There are different classifications available, and each country or programme uses the one most appropriate to their setting.

The retinal features of DR recorded by the graders include: microaneurysms, hard exudates, retinal haemorrhages, cotton wool spots, venous changes, blot haemorrhages, intraretinal microvascular abnormalities, pre-retinal fibrosis, new vessels and tractional retinal detachment.

